# Neutrophil Extracellular Traps: A Potential Therapeutic Target in MPO-ANCA Associated Vasculitis?

**DOI:** 10.3389/fimmu.2021.635188

**Published:** 2021-03-15

**Authors:** Kim M. O'Sullivan, Stephen R. Holdsworth

**Affiliations:** ^1^Department of Medicine, Centre for Inflammatory Diseases, Monash University, Clayton, VIC, Australia; ^2^Department of Nephrology, Monash Medical Centre, Clayton, VIC, Australia; ^3^Department of Immunology, Monash Medical Centre, Clayton, VIC, Australia

**Keywords:** ANCA, glomerulonephritis, cell death, NETs, MPO

## Abstract

Our understanding of immune recognition and response to infection and non-infectious forms of cell damage and death is rapidly increasing. The major focus is on host immunity and microbiological invasion. However, it is also clear that these same pathways are important in the initiation and maintenance of autoimmunity and the damage caused to targeted organs. Understanding the involvement of cell death in autoimmune disease is likely to help define critical pathways in the immunopathogenesis of autoimmune disease and new therapeutic targets. An important immune responder cell population in host defense and autoimmunity is the neutrophil. One autoimmune disease where neutrophils play important roles is MPO-ANCA Microscopic Vasculitis. This a severe disease that results from inflammation to small blood vessels in the kidney, the glomeruli (high blood flow and pressure filters). One of the best studied ways in which neutrophils participate in this disease is by cell death through NETosis resulting in the discharge of proinflammatory enzymes and nuclear fragments. In host defense against infection this process helps neutralize pathogens however in auto immunity NETosis results in injury and death to the surrounding healthy tissues. The major autoimmune target in this disease is myeloperoxidase (MPO) which is found uniquely in the cytoplasm of neutrophils. Although the kidney is the major organ targeted in this disease MPO is not expressed in the kidney. Autoantibodies target surface MPO on activated circulating neutrophils resulting in their lodgment in glomerular capillaries where they NETose releasing extracellularly MPO and nuclear fragments initiating injury and planting the key autoantigen MPO. It is the cell death of neutrophils that changes the kidney from innocent bystander to major autoimmune target. Defining the immunopathogenesis of this autoimmune disease and recognizing critical injurious pathways will allow therapeutic intervention to block these pathways and attenuate autoimmune injury. The insights (regarding mechanisms of injury and potential therapeutic targets) are likely to be highly relevant to many other autoimmune diseases.

## Introduction

Anti Neutrophil Cytoplasmic Antibody (ANCA) associated vasculitis (AAV) is an autoimmune disease characterized by inflammation of the small blood vessels. AAV can be divided into three different clinical categories: Microscopic polyangiitis (MPA), Granulamotisis with polyangiitis (GPA), and Eosinophilic granulomatosis with polyangiitis (EGPA). A common clinical feature between all 3 vasculitides is a loss of tolerance to neutrophil enzymes, myeloperoxidase (MPO), and proteinase 3 (PR3) which results in the generation of ANCA to either MPO or PR3. MPA patients have a high incidence of MPO-ANCA development and is more prevalent in the Asia pacific region. GPA is the dominant clinical subtype of AAV north of the equator. GPA patients have a higher incidence of PR3-ANCA.

The major organ effected is the kidney (more frequently observed in MPA disease) often resulting in rapidly progressive crescentic glomerulonephritis (RPCGN), which has the worst outcome. If left untreated 90% of patients will progress to renal failure and eventual death. Patients present with general fatigue, elevated temperature, flu-like symptoms accompanied with weight loss and diminished appetite (1, 2). These symptoms result from systemic inflammation and loss of kidney function. Extra renal symptoms are common and often characteristic and particular to their specific disease.

Until the introduction of nitrogen mustard based immunosuppression most notably cyclophosphamide, treatment was primarily based on high doses of glucocorticoids. Prior to this regimen most patients would progress to end stage renal failure within 5 months (3–6). With discovery of ANCA as a diagnostic marker in the 1980s and with current treatment, remission can now be induced within 6 months and survival rates have increased to approximately 75% of patients at 5 years. Although these treatment regimens are largely successful, relapse rates remain high at around 50% at 5 years. Despite maintenance therapy, the high percentage of relapses suggest that standard treatment does not actually completely delete the pathogenic autoimmunity. The toxicity of the treatment itself contributes significantly to morbidity and mortality (3–6). Drug-related toxicity and adverse effects will cause difficulty in over 90% of patients. The most common form of death within the first year of contracting the disease will be infection associated with the immunosuppressed state of the patient. Prolonged use of cyclophosphamide is also associated with an increase rate of malignancy (7).

There is an unmet need for more specific treatment to treat the underlying immunopathology in AAV. Teasing out the underlying pathogenic mechanisms of directing AAV is essential for the development of effective treatment strategies. A key pathological feature of MPO-AAV is the accumulation of dying neutrophils. Neutrophil cell death releases the key autoantigens in ANCA associated vasculitis (AAV); myeloperoxidase (MPO) and proteinase 3 (PR3). Alongside MPO and PR3 one of the key molecules released during cell death is double stranded deoxyribonuclease acid (dsDNA). Without the protection of the cell membrane dsDNA activates multiple sensors and signaling pathways, amplifying inflammation. This review will evaluate the contributing role of Neutrophil Extracellular Traps (NETs) to the pathogenesis of MPO-AAV, with a focus on the activation of NETs, the deposition of NET derived antigens and danger associated molecular patterns (DAMPS) and the development of new experimental NET inhibitors which show promise in attenuating pathological damage to the kidney.

## Overview of the Pathology of ANCA Associated Vasculitis

**Figure 1:**
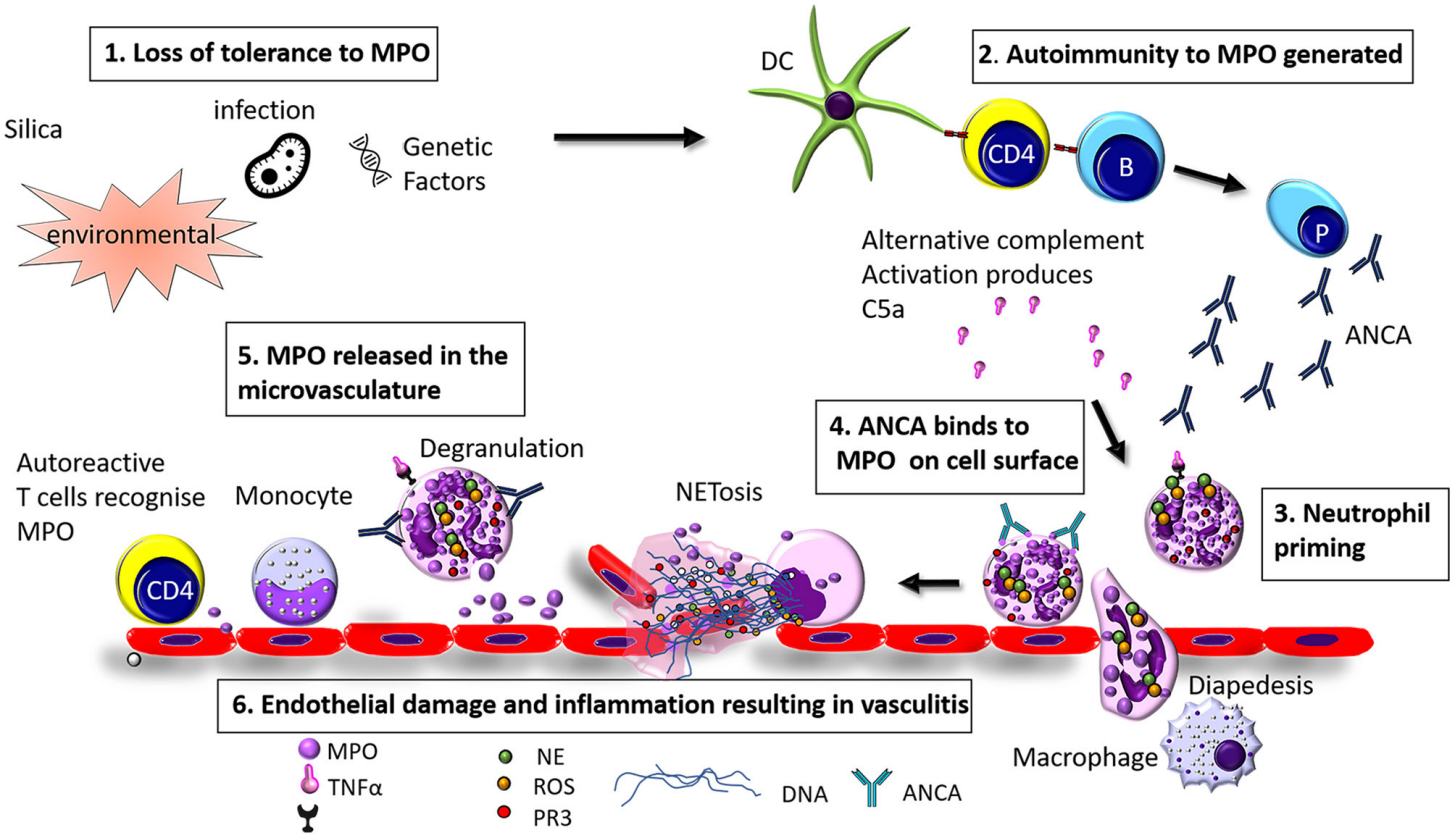
Hypothesized pathogenesis of Myeloperoxidase anti neutrophil cytoplasmic antibody vasculitis. (1) Loss of tolerance of MPO can be caused by infection, environmental factors (such as exposure to silica), or genetic factors (2) Loss of tolerance to MPO results in the production of MPO-ANCA and anti MPO specific T cells (3) Neutrophils are primed by inflammatory mediators such as TNFα, LPS, IL-8, or C5a (4) ANCA binds to MPO exteriorised on the cell surface and (5) MPO is released in to the glomerular vasculature through degranulation or NETosis (6) Endothelial cells are injured resulting in inflammation and vasculitis.

AAV is a disease of unknown etiology. The pathogenesis of AAV is complex and involves a wide range of pathogenic processes. In the case of MPO-AAV and PR3-AAV there is a loss of immunological tolerance to neutrophil enzymes MPO and PR3, this in turn generates ANCA and MPO specific T cells. Neutrophil priming and activation via C5a and C5aR interactions exteriorise MPO (or PR3) on their cells surface which MPO-ANCA (or PR3 ANCA) binds to, these neutrophils home to the microvasculature of the glomeruli via selectins, P, E, and L which play a role by reducing the speed of the neutrophil in the circulation which allows it to roll. The second signal comes from the neutrophil surface integrins β_1_ and β_2_ which interact with the ICAM-1 and ICAM-2 ligands on the inflamed endothelium, the rolling neutrophils slow down, and crawl along the endothelium mediated by integrins to the site of transmigration from the capillaries into the interstitium. Neutrophils either degranulate or release neutrophil extracellular traps (NETs, to be discussed in detail in a later section), causing direct injury through the release of enzymes, reactive oxygen species (ROS), and proteases. Excess neutrophils, MPO specific T cells, and macrophages [via a delayed type hypersensitivity (DTH) mechanism] are recruited resulting in a vicious cycle of injury which causes glomerular injury (8) (see Figure 1).

## Neutrophil Activation in MPO-AAV

The first *in vitro* experiments demonstrating the potential pathogenicity of ANCA came from the laboratory of Falk et al., where it was demonstrated that neutrophils can be activated by ANCA (9). Purified ANCA sera from patients' with pauci-immune necrotizing and crescentic GN incubated with normal human neutrophils, induced a reactive oxygen species burst (ROS) followed by degranulation of the neutrophil. Further flow cytometry studies demonstrated that after priming neutrophils with the cytokine tumor necrosis factor (TNF) neutrophils exteriorized MPO to the cell surface. The surface location of MPO allowed interaction with ANCA.

Brouwer et al. (10) further demonstrated both *in vitro* and *in vivo* that the numbers of activated neutrophils within kidney biopsies of patients with Wegener's granulomatosis correlated significantly with serum creatinine levels taken at the time of biopsy (22 PR3 positive and 5 MPO positive) (10). All ANCA samples from patients (*n* = 19) were capable of activating primed neutrophils, however no correlation was observed between the ANCA titer of patients and the numbers of activated neutrophils within renal biopsies. A major deficiency of the hypothesis that ANCA induced renal disease was the fact that the key autoantigens, MPO and PR3 were not expressed in the kidney, the major organ damaged by autoimmunity to these antigens. This was also the first study to demonstrate the presence of neutrophil enzymes MPO, PR3 and elastase (HLE) extracellularly within renal biopsies. Their deposition occurred when neutrophils were trapped in the glomerular capillaries where degranulation and other forms of neutrophil cell death released these autoantigens extra cellularly into the kidney. This initiates injury, attracting anti MPO/PR3 T cells driving Delayed Type Hypersensitivity (DTH) effector responses which also induces further neutrophil attraction. In summary neutrophils play an essential role in converting the kidney from innocent bystander status to secondary target status where deposited MPO within the kidney is recognized as an antigen by MPO specific T and B cells.

A major advance in confirming the unique pathway and role of renal targeting by neutrophils was an experiment by the Falk and Jennette group using neutrophil depleted mice. These mice could develop anti-MPO autoimmunity but they could not develop GN unless neutrophils were infused into their circulation. Exogenous neutrophil infusions allowed the development of anti MPO vasculitis and GN confirming the non-redundant role played by neutrophils in the disease (11, 12).

Studies in human ANCA demonstrated that ANCA IgG binds to the MPO or PR3 expressed on the surface of human neutrophils and ligates the Fragment Crystalline gamma RIIa receptor (FCγRIIa). Porges et al. demonstrated that blockade of the FCγRIIa with a monoclonal antibody significantly reduced the production of ROS of ANCA activated neutrophils (13). Binding of ANCA to the FCγRIIa receptor may be one mechanism in which human neutrophils activate signal transduction pathways which result in inflammation (13).

In addition to FCγRIIa other studies have also shown a role for FcγRIIIb in ANCA activation of neutrophils. Kocher et al. demonstrated that FCγRIIa requires a high density of ANCA binding whereas FcγRIIIb is expressed at 10x higher density than FcγRIIa on the cell surface of neutrophils (14). These results are indicative of FcγRIIIb being involved in the initial engagement by ANCA and that cross linking of the FcγRIIIb favors adhesion of activated neutrophils to the endothelium.

Further *in vitro* studies demonstrated the role of cytokines in priming neutrophils for ANCA activation. Kettritz et al. further confirmed the requirement of cytokine priming of neutrophils with TNFα showing the translocation of both MPO and PR3 to the cell surface. Studies of both the binding and activating properties of ANCA, indicated that both intact ANCA and the F(ab)_2_ portion of the Ig could cross link MPO or PR3 on the membrane surface of neutrophils (15). ANCA binding alone was not sufficient enough to activate neutrophils, crosslinking of either MPO or PR3 on the cell surface was required to induce a ROS burst.

### Complement Activation of Neutrophils in AAV

The complement system is comprised of 3 distinct pathways, the classical, lectin, and alternative pathway all of which produce effector molecules used to fight infection. All 3 pathways initiate a complement cascade converging to form C3 and C5 convertases. The resulting complement cascade generates C5a which binds to the C5aR1 and C5aR2. C5aR1 is expressed on multiple immune cells, including neutrophils and monocyte/macrophages. Cleavage products of the complement cascade (C3a and C5a) are seen by the immune system as danger signals, and recruit further cells of the immune system, in particular neutrophils.

In the context of AAV, evidence suggests that C5a-C5aR interactions are pathological. C5a can prime neutrophils for NET formation, recruit neutrophils to the vascular bed of the glomeruli, and enhance Dendritic cell activation (16). Multiple animal studies of AAV have shown that the alternative pathway plays a critical role in priming neutrophils for activation by ANCA via signaling through C5a-C5aR1 interactions (16–18). Complement activation factors are increased in patients with active AAV disease. These observations suggested that C5a may be a therapeutic target in AAV. Inhibition of the C5aR1 with the antagonist CCX168 (Avacopan) has been demonstrated in phase II and III clinical trials to be effective in modifying disease severity in patients with active AAV (19–22).

## Neutrophil Extracellular Traps (NETs)

A novel mechanism by which neutrophils kill microorganisms was discovered by Brinkmann et al. (23). This study demonstrated that activated neutrophils were able to produce extracellular traps containing DNA fibers, coated with neutrophil proteins from the primary granules (MPO, NE, cathepsin G), secondary granules (lactoferrin), tertiary granules (MMP9), and histones (H1, H2A, H2B, H3 and H4 and the H2A-H2B DNA complex). This seminal study suggested that neutrophils produce NETs to amplify the effectiveness of their antimicrobial granules by producing a large net in a concentrated area, which formed both an antimicrobial and physical barrier to prevent the spread of microorganisms. The authors of this study very insightfully suggested that this mechanism may also have a detrimental effect on the host that could stimulate autoimmunity (23). This initial work, opened a new field in neutrophil biology that in the last 16 years has expanded to define, the signaling pathways for NET production, identification methods of NETs, and a role for NETs in perpetuating inflammation in autoimmune diseases. NETosis is a powerful inducer of injury and therefore a major potential therapeutic target.

### Sequence of Events Required for NETosis

**Figure 2:**
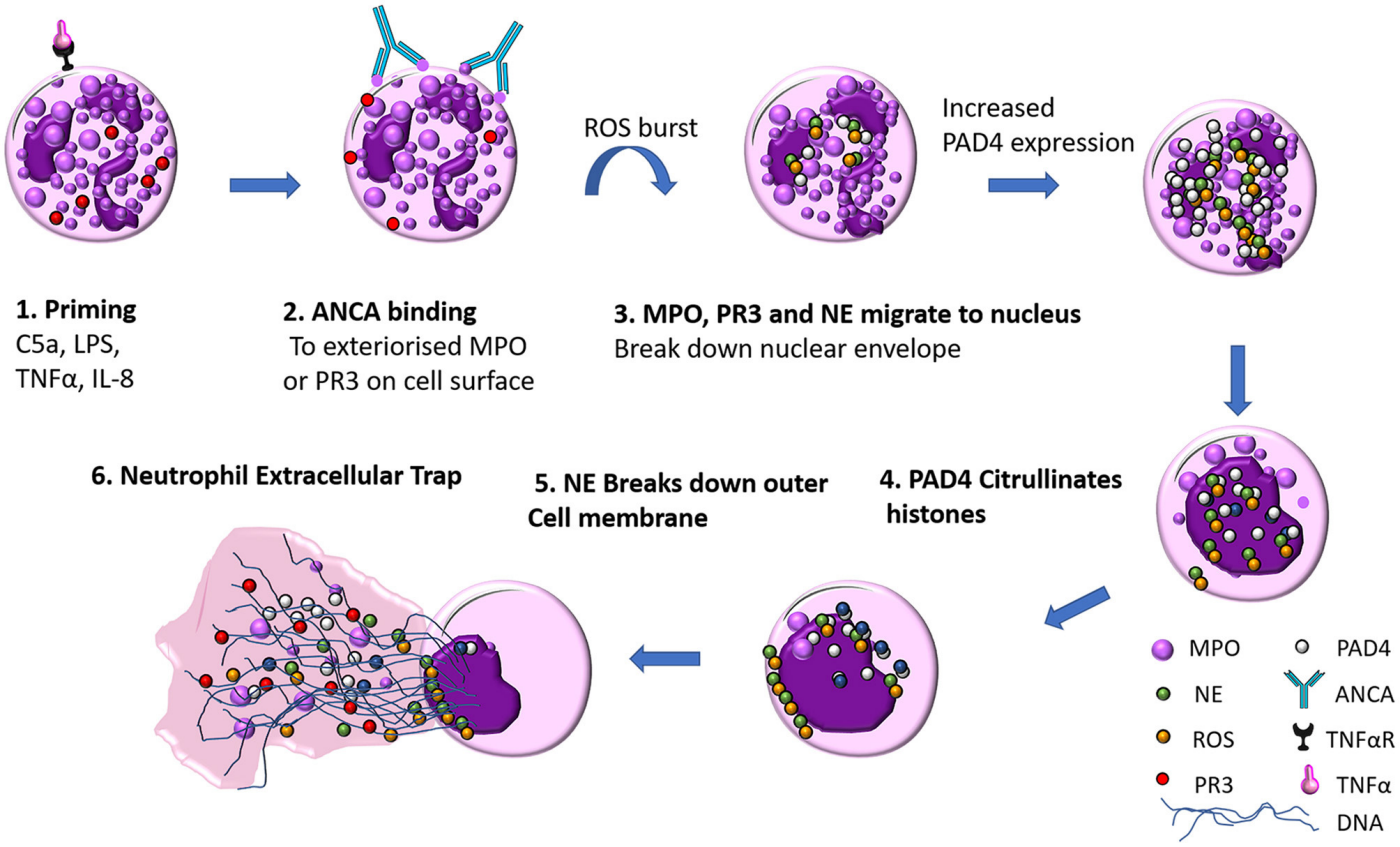
ANCA mediated neutrophil extracellular trap (NET) formation. (1) Neutrophils are primed by inflammatory mediators (such as TNFα, LPS, IL-8, or C5a), which exteriorises MPO on the cell surface (2) ANCA antibody recognize MPO as their cognate antigen and bind to the surface of the neutrophil which initiates a ROS burst (3) NE, PR3, and MPO migrate to the nucleus where they break down the nuclear envelope (4) Increased PAD4 expression facilitates citrullination of histones, the contents of the nucleus and cytoplasm mix as DNA decondenses (5) Neutrophil elastase breaks down actin fibers of the neutrophil cytoskeleton (6) DNA is released with MPO, PR3, ROS and citrullinated histones.

NET formation is a unique form of neutrophil cell death that it is not instigated by either necrosis or apoptosis. Live cell imaging of NETs by Fuchs et al. revealed the basic steps required for NET formation (24). PMA stimulated peripheral neutrophils from healthy donors were imaged for 240 min, and the steps in NET formation were outlined to follow a pattern: Firstly 60 min after stimulation, neutrophil nuclear lobules begin to lose their shape, the nuclear envelope begins to disintegrate into small vesicles and chromatin decondensation begins with segregation of eu- and heterochromatin, 180 min after stimulation the nuclear envelop has completely disintegrated, granular membranes rupture and the decondensed chromatin mixes freely with both the contents of the granules and cytoplasmic material, then the outer cell membrane ruptures and a protrusion of a net like structure comprised of DNA, histones, MPO, and NE is extruded (see Figure 2). The authors through elegant experiments determined that this form of cell death was neither apoptosis nor necrosis as DNA fragmentation a key marker in apoptosis was absent via TUNEL staining in NET formation. Necrotic cells did not make NETs, their nuclear membranes did not rupture, and the nucleus just fused into a homogenous mass with no apparent segregation of eu- and heterochromatin (24).

### NETosis Requires ROS

Reactive oxygen species (ROS) are required for the formation of NETs. The most compelling evidence comes from patients with Chronic Granulomatous Disease (CGD) who have genetic deficiencies that cause mutations in nicotinamide adenine dinucleotide phosphate (NADPH) oxidase. CGD patients are unable to form ROS and as a result are unable to make NETs (24). CGD patients suffer from recurrent bacterial and fungal infections that they are unable to clear infection efficiently due to the lack of NADPH which is required for phagocytosis by granulocytes. Addition of H_2_O_2_ to CGD patients' neutrophils *in vitro* enables neutrophils to produce NETs, suggesting that the sequence of events that allows NET formation can be restored downstream of NADPH (24).

### Role of Peptidyl Arginine Deiminase (PAD4) in the Citrullination of Histones

One of the determining factors that distinguishes between neutrophil NET formation and either apoptosis or necrosis is the citrullination of histones mediated by PAD4. Initial observations of the role of PAD4 and its association with deimination of histones in neutrophils came from experiments in animal models of rheumatoid arthritis where the expression of PAD4 and citrullinated histones correlated with an increase in disease severity (25). Neeli et al. observed in experiments with stimulated human neutrophils that NET formation was associated with deimination of H3, whereas apoptotic cells produced no deimination of H3, making citrullination of H3 a novel marker to distinguish between NET formation and apoptosis (26). To investigate further the requirement of PAD4 in the citrullination of histones in NET formation Wang et al. demonstrated that the inhibition of PAD4 by the pan PAD inhibitor Cl-amidine failed to produce NETs in HL-60 granulocytes, through the inhibition of H3 citrullination (27). The same group followed up these experiments in PAD4^−/−^ mice and demonstrated that PAD4 was required for NET mediated efficient bacterial killing. Neutrophils stimulated to form NETs by LPS, PMA, and H_2_O_2_ *in vitro* all required PAD4. PAD4^−/−^ neutrophils were unable to form NETs in response to any of the stimuli. Using a model of necrotizing fasciitis the authors demonstrated that PAD4^−/−^ mice were unable to produce NETs and had a reduced capacity to clear bacterial infection compared with PAD4+/+ mice (28).

### Critical Enzymes and Molecules Required for NETosis

Both NE and MPO are stored in the primary (azurophilic granules) of neutrophils, and are both found abundantly adhered to NET fibers. Experiments by Papayannopoulos et al. demonstrated that translocation of NE to the nuclear envelope was required for the decondensation of chromatin (29). After the initial ROS burst NE is released from primary granules where it migrates to the nucleus and digests nucleosomal histones, ultimately instigating the decondensation of the chromatin. MPO was also found to bind to chromatin and enhance decondensation independent of its enzymatic functions. The role of MPO in NET formation was further explored in subsequent experiments and neutrophils from 100% MPO deficient individuals were shown to be unable to form NETs (30). Interestingly neutrophils from individuals with only a partial MPO deficiency were still able to make NETs. MPO contributes 5% of the dry weight of neutrophils and is the most abundant neutrophil granzyme. MPO generates hypochlorous acid through the oxidation of chloride and halide ions in the presence of H_2_O_2_, this is highly antimicrobial. Despite the requirement of MPO to generate NETs and its highly effective antimicrobial properties, a genetic deficiency in MPO does not appear to have a major impact on individuals with this defect, they generally live healthy lives unless they have co-morbidities such as diabetes (31).

The importance of MPO in regulating ROS was determined by demonstrating that MPO inhibitors successfully blocked PMA induced NET formation, and that this process could not be rescued downstream by adding extracellular MPO (32). Observations by other laboratories suggest the requirement for MPO in NET formation may be dependent on the activating stimulus. Neither ROS nor MPO is required for NET formation when triggered by *Leishmania* parasites, but NE was required (33). These findings were in agreement with experiments by Parker et al., who found that MPO was required for NET formation when stimulated by PMA but not by *S. aureus* and *E. coli* (34). Overall these experiments highlight the need for physiological relevant experiments, although PMA is a potent NET inducer, it is not found physiologically in either humans or mice and caution should probably be used when using it to study signaling pathways, and assessing requirements for enzymes and ROS, as they may not be representative of what happens *in vivo*.

### Role of Gasdermin D in NET Formation

Pyroptosis is a lytic form of inflammatory cell death where Gasdermin D (GSDMD) plays a critical role in forming pores that allow the influx of water resulting in swelling of the cell and osmotic lysis (35). Recently GSDMD has been implicated in having a crucial role in the formation of NETs.

During a large screening of small molecule inhibitors of NETosis Sollberger et al. identified a compound LDC7559 with a pyrazolo-oxazepane scaffold that binds to GSDMD preventing NETosis (36). Using LDC7559 to inhibit NET formation it was demonstrated that neutrophil elastase (NE) and GSDMD work together to break down the nuclear envelope and again to break down the cell membrane at point of expulsion of the DNA webs. LDC7559 inhibits NET formation at low concentrations (1 μM) without blocking phagocytosis, ROS production, NE or MPO activity. These attributes of LDC7559 have potential as therapeutic treatment of diseases with excessive NET burden as it will prevent NETosis whilst keeping host defense intact. This work further consolidated the critical role of NE in NETosis as NE is essential for the activation of GSDMD. This highlights both NE inhibitors and GSDMD as potential therapeutic benefit in inhibition of NETs in inflammatory diseases (29, 30, 36).

Amongst the first hypothesized steps in NETosis we can now add the role of GSDMD (24). The accepted chain of events are: (1) the neutrophil undergoes a ROS burst, followed by (2) migration of neutrophil enzymes NE and MPO to the nucleus where NE induces break down of the nuclear envelope via cleavage and activation of gasdermin-D that punches holes in the nuclear membrane, (3) increased PAD4 expression facilitates citrullination of histones and decondenses DNA, (4) NE destabilizes the structure of chromatin and modifies histones and cleaves actin within the cytoskeleton weakening the outer cell membrane to allow, (5) expulsion of the NET.

### Caspase 11 Driven NETosis

Traditionally defined NETosis requires citrullination of histones via PAD4, neutrophil elastase and to a limited extent MPO. A recently discovered form of NETosis driven by detection of cytosolic LPS activates caspase 11 and occurs independently of the requirement of traditional neutrophil enzymes. Inhibition of MPO, NE and PAD4 does not inhibit caspase 11 mediated NETosis (37). This mechanism is thought to have evolved to enhance gram negative bacteria killing by releasing antimicrobial NETs of DNA. Inhibition of both GSDMD and caspase 11 blocks NETosis. Release of MPO and NE is inhibited, which suggests that GSDMD is most likely to be involved in creating pores in the membranes of lysosomes which are responsible for liberating MPO and NE (37). Caspase 11 and GSDMD work in partnership to facilitate NETosis in two stages of NETosis. In stage 1 GSDMD creates pores in the nuclear membrane allowing caspase 11 to enter and cleave histones allowing chromatin relaxation for NET extrusion. In stage two GSDMD creates pore in the cell membrane to allow release of the DNA (37).

Although this type of NETosis is thought to have evolved to defend neutrophils against the invasion of gram negative bacteria, there are other implications where this may be relevant in other diseases. Both GSDMD and caspase 11 may be potential therapeutic targets.

### Signaling Pathways in NET Formation

Several different pathways have been reported to play functional roles in NET formation. The first of these pathways is known as the Raf (rapidly accelerated fibrosarcoma),- MEK-(mitogen-activated protein kinase), -ERK (extracellular signal-regulated kinase pathway). The Raf-MEK-ERK pathway is responsible for controlling the expression of the anti-apoptotic protein Mcl-1. PMA stimulated neutrophils downregulate Mcl-1 immediately after stimulation, which blocks apoptosis and favors NET formation. PKC, cRaf, and MEK inhibitors can block this downregulation, suggesting that the raf-MEK-ERK pathway is responsible for controlling NET formation. The authors of this study suggest this pathway could be a potential therapeutic target in diseases where aberrant NET formation occurs (38).

There has been speculation that NET formation could also be activated by the same pathway that causes necroptosis in neutrophils. Necroptosis is another form of cell death distinct from apoptosis. It is considered to be a programmed form of necrosis. The simplified version of the complex signaling pathway is that once activated a series of signals cause interaction between RIPK3 (receptor interacting protein kinases-3) which phosphorylates MLKL (mixed lineage kinase domain-like), which creates a conformational structural change allowing the oligomerization of MLKL to form a pore, which results in increased osmotic pressure within the cell as ion and water enter the cell resulting in rupture of the cell membrane. Experiments using *Ripk3*^−/−^ mice and MLKL inhibition demonstrated that this pathway is used downstream of ROS production (39). Nec-1 an inhibitor of necroptosis, which prevents the formation of the necrosome through the modulation of RIPK1 and the subsequent phosphorylation of RIPK3 and MLKL, prevented the formation of NETs induced by PMA or monosodium urate (MSU) crystals in both human and mouse neutrophils. In direct contrast, Amini et al. found that NET formation occurred independent of signaling through RIPK3 and MLKL (40). Using the neutrophils from the genetically modified mice deficient in *Ripk3* and human neutrophils chemically inhibited with the MLKL inhibitor necrosulfonamide (NSA), the authors conclude that NET formation is independent of a necroptotic form of death. The notable difference between these 2 studies could be dependent on the different stimuli used to activate and stimulate NET formation. Desai et al. used MSU crystals, and PMA whereas Amini et al. used GM-CSF primed neutrophils with C5a, LPS, or *E. coli*.

ANCA mediated NETosis can be mediated by the RIKP1/3 MLKL pathway (22). *In vivo* NETs can be pharmacologically inhibited using the RIPK1 inhibitor Nec-1s, and the MLKL inhibitor necorusulfonamide (22). RIPK3 and MLKL deficient mice are protected from the development of necrotizing crescentic glomerulonephritis induced by anti-MPO antibody. RIPK3 deficient bone marrow (BM) transfers, in the same animal model were protected suggestive of necroptosis playing a critical role in the progression of necrotizing crescentic glomerulonephritis.

### MPO Is Biologically Active in NETs

The bactericidal capacity of MPO in NETs has been shown *in vivo* to be biologically active when adhered to DNA remnants from NET formation. Parker et al. measured the peroxidase activity of MPO released from NETs, and found that over 80% of the MPO released from the neutrophil came from NET formation, and was still biologically active (34). The findings of these experiments have two implications. Firstly, MPO adhered to NETs may aid in the bactericidal capacity of NETs and increase the role of neutrophils role in reducing the spread of bacteria at sites of infection. The second implication, is that the extracellular NET MPO that is deposited is biologically active and may cause direct injury to the surrounding tissues and also become available as an autoantigen in autoimmunity in AAV.

### NETs Transfer Neutrophil Antigens in AAV

Although the role of NETs in enhancing host defense against bacterial infection has been well-established, the production of NETs also has potential to expose the immune system to potential self-antigens, and therefore may play a role in perpetuating inflammation by triggering and enhancing injurious autoimmunity to extracellular MPO.

Proof of concept that NETs can initiate autoimmunity in AAV comes from animal models of the disease. Sangaletti et al. demonstrated that neutrophil antigens from NETs can be transferred to dendritic cells (DCs) (41). Live cell imaging of stimulated pro inflammatory neutrophils co-cultured with myeloid DCs (mDCs) showed that NET forming neutrophils make stable connection with mDCs, whilst apoptotic neutrophils only form infrequent contact with mDCs. To examine the possibility of the transfer of the auto-antigens MPO and PR3 from dying neutrophils to mDCs, co- cultures of neutrophils and mDCs were observed and the efficacy of neutrophil antigen transfer characterized. Necrotic cells failed to transfer either MPO or PR3. These auto-antigens were internalized by the DCs with apoptotic bodies less frequently than MPO or PR3 from NET forming neutrophils. Addition of DNase I to the co-culture media prevented the transfer of NET associated antigens, by disintegrating the DNA back bone of the NETs, indicating that the intact DNA structure is required for successful transfer of the autoantigens. Based on these findings the authors of this study transferred the mDCs cultured with either NETs or apoptotic neutrophils into mice via intraperitoneal injection, weekly for a period of 6 weeks, collected serum and examined both the kidneys and lungs for pathology at the termination of the experiment 4 months later. Results demonstrated that the mDCs co-cultured with the NET forming neutrophils had an increased production of circulating ANCA, IgG, and C3 deposition, and an increased kidney pathology score compared to the apoptotic neutrophils co-cultured with mDCs, which had a reduced amount of ANCA production and no evidence of vasculitis in either the lungs or kidneys.

This study provided proof of concept that NET forming neutrophils can initiate vasculitis. The authors conclude that AAV skews neutrophil cell death toward NETosis rather that apoptosis. They also confirm that apoptotic cell death is more likely to protect from excessive inflammation and the development of autoimmunity. The authors do not discuss the possibility that apoptotic neutrophils co-cultured with mDCs may actually have a protective effect. This has been reported in other diseases such as type 1 diabetes (42). DCs co-cultured with apoptotic bodies from β cells and injected into mice have been shown to induce a reduction in co-stimulatory molecules and production of cytokines in NOD diabetic mice (42).

NETs not only exposed the autoantigens MPO and PR3 but also make histones available for interaction with the immune system. Histones themselves elicit strong responses from the immune system by activating danger associated molecular patterns (DAMPS), recognized in particular by toll-like receptors (TLR) 2, TLR4, and nucleotide-binding oligomerisation domain like (NOD) receptors. Histones have been implicated in enhancing injury in many other forms of disease such as sepsis, trauma associated lung injury, sterile liver injury, and kidney injury (43–47).

Histone capacity to induce inflammation and injury is supported by the actions of PAD4. A recent study by Kumar et al. found that inhibition with the PAD inhibitor Cl-amide or histone antibody depletion, reduced glomerulonephritis in a mouse model of anti GBM (48). A reduction in glomerular crescents, and leukocyte infiltration was significant, indicating that targeting extracellular histones could be an effective treatment in glomerulonephritis.

### NET Interactions With T Cells

NETs have an established role in innate immunity, but less is known about their interactions with the adaptive immune system. *In vitro* studies of human NETs and T cells have indicated that NETs can prime CD4+ T cells by decreasing their activation threshold (49). Co-culture of T cells and NETs alone increases the upregulation of the T cell activation markers CD25 and CD69, but does not increase T cell proliferation. Co-cultures of DCs, T cells and NETs, results in an increase in the activation of T cells, measured by the increased production of T cell proliferation cytokines IFNγ, IL-17A, IL-4, IL-10, and IL-2. This effect however is not seen when NETs alone are cultured with T cells, or in transwell plates where the DCs and NETs are separated from the T cells, indicating that T cell direct contact with both DCs and NETs is required for T cell activation (49).

*In vivo* studies of NETs indicate they can activate Th17 cells, indirectly by priming human macrophages and in a murine model of atherosclerosis (50). Co-cultures of monocytes with NETs activated by cholesterol crystals resulted in an increased production of monocyte cytokines (including IL-1β). In a model of murine atherosclerosis, NETs were found associated with atherosclerotic lesions, which could be reduced when disease was triggered in NET deficient mice (APoE, PR3, and NE deficient) to prevent NET formation. The production of IL1β by macrophages increased the secretion of T cell derived IL17, and subsequent neutrophil recruitment to vascular endothelia (50).

### Complement and NET Interactions

Neutrophils from either C3^−/−^ or C3a receptor^−/−^ (C3aR) mice are unable to form NETs (51, 52). This indicates that complement is non-redundant in initiating NETosis. Activated neutrophils express complement factors such as C3 and properdin and Factor B on their cell surface- all components of the alternative complement pathway (53). In host defense neutrophils form NETs to increase bactericidal killing by increasing their surface area in which they ensnare bacteria. Complement opsonization of bacteria have an enhanced ability to induce NETosis (54). C5a both recruits' neutrophils and primes them for NET induction through upregulation of Toll-like and complement receptors (55). Neutrophil enzymes contained with the DNA fibers of NETs have the capacity to cleave C3 and C5 into active fragments. For example MPO can cleave C5a and C5b, whereas Neutrophil Elastase can cleave C3 in to C3a and C3b (56, 57).

## Observations of NETs in Clinical AAV

Kessenbrock et al. provided the first data that indicated a possible pathological role for NETs in AAV. ANCA IgG from patients with small vessel vasculitis (SVV) were able to trigger NET formation in isolated human neutrophils from healthy individuals primed with TNFα (23% of neutrophils formed NETs), whereas IgG from normal healthy individual was only able to stimulate 11% of neutrophils to form NETs. The main autoantigen targets MPO and PR3 were both present upon the NET DNA fibers and were still accessible for ANCA Ig to bind to. After establishing that ANCA could activate NET formation and that the auto antigenic targets MPO and PR3 were accessible within the NETs the authors sought evidence of *in vivo* NET formation in the biopsies of AAV patients. Using antibodies to NET components (DNA, histones, MPO, NE, PR3, and LL37), they found that NETs were present in 9/15 biopsies examined (58).

We have demonstrated that NET formation is closely associated with the deposition of MPO in glomeruli in a large clinical study of MPO-AAV patients (*n* = 47) (59). Extracellular MPO was significantly higher in glomeruli with NETs. This was also the first study to demonstrate the presence of macrophage extracellular traps (METs) that were MPO positive and frequently observed within the glomeruli of MPO-AAV patients (59). Considering macrophages are one of the prevalent leukocyte subtypes infiltrating glomeruli in AAV biopsies, potential therapies targeting NETs should also extend to METs.

Further studies of NET involvement in AAV patients include case reports demonstrating NET formation through the use of antibodies against MPO, Citrullinated Histones and PAD4 or using DAPI in combination with MPO to identify NETs (60, 61). A larger study containing 15 AAV patients (for peripheral neutrophil studies) and 6 kidney biopsies in China showed similar results to the Kessenbrock et al. study, demonstrating enhanced NET formation in patients with AAV compared to healthy controls. There was evidence of increased NET formation in the biopsies of patients with active crescentic GN but minimal NET evidence in the non-crescentic patients providing evidence that NETosis maybe injurious.

Attempts have been made by several research groups to show an association between circulating NET markers and active disease. In a large cohort of 93 AAV patients Soderberg et al. (62), found that patients with active AAV had significantly more remnants of NETs than healthy controls within their circulation. No difference was found between patients in remission and healthy controls. In contrast, Wang et al. found the level of circulating Neutrophil extracellular traps did not correlate with disease activity. In this cohort there were 34 patients with active AAV and 63 patients with AAV in remission (63). A significant difference in the numbers of circulating NETs between AAV patients and healthy controls was determined using circulating cell free DNA (cfDNA) as a marker of NETs. There was no correlation between NET formation in the circulation of patients with active disease when compared to patients in remission.

Wang et al. investigated the levels of DNase I an enzyme that specifically degrades DNA and found that patients with AAV had significantly higher circulating levels of DNase I than healthy controls whereas there was no observed difference between the active AAV and remission group. Taken together current data does not suggest that the presence of NET remnants in the serum is a reliable indicator for disease severity or activity (63).

More recently it has been shown that NET formation can occur in AAV independent of ANCA (64). In a cohort of 99 AAV patients, patients' neutrophils isolated from whole blood demonstrated excessive NET formation *ex vivo* when stimulated with ANCA serum depleted of IgG. Serum levels of ANCA did not correlate with NET remnants, however increased NET formation was observed in patients with active disease. Of interest patients with MPO-AAV had significantly more NET formation than those of PR3-AAV patients. Neither C5aR inhibitors or eculizumab were able to prevent formation of NETs *ex vivo*, indicating that C5a-C5aR interactions are not critical for human NET formation *ex vivo*. Whereas, a genetic deficiency in either C3a or C3aR prevents NET formation in murine neutrophils (as discussed earlier).

NET formation in AAV is distinct from NET formation observed in systemic lupus erythematosus (SLE). Serum from *n* = 80 AAV patients and *n* = 59 SLE patients, were incubated with healthy circulating neutrophils, and induced excessive NET formation. AAV patient serum significantly induced more NETs compared to serum from both healthy controls and SLE patients (65). The qualitative appearance of NETs from AAV patients also differed from SLE patients. AAV induced NETs were lytic in nature with large expulsion of DNA, and occurred much slower than that of SLE induced NETs which were faster, with less expelled DNA, a higher concentration of mitochondrial DNA (mtDNA) and less lysis of the cell membrane. These results should be interpreted with caution as they were performed *ex vivo* by incubating patient serum with healthy neutrophils. A more efficient way of determining the difference between AAV and SLE NETs would be to conduct the same experiment with the neutrophils from the patients. As previously mentioned AAV neutrophils have a higher propensity to NET than those of healthy neutrophils, therefore data collected from neutrophils form healthy patients and SLE patients may not behave in the same manner (62).

Collectively these disparate results indicate using circulating NETs is not a good biomarker for AAV disease activity. Observations from *ex vivo* experiments may not be representative of what happens *in vivo*. Detection of NETs in kidney biopsies would provide more relevant evidence of pathogenicity as these are the sites of direct injury opposed to measuring NET remnants from the circulation in serum, or inducing NET formation *ex vivo*.

## Vital NETs and Mitochondrial DNA Release

Neutrophils are also able to produce NETs which do not ultimately end in death of the neutrophil. This has been termed “vital NETosis,” and uses some of the same pathways as traditional “suicidal NETosis,” such as the translocation of NE to the nucleus, activation of PAD4 and decondensation of chromatin. Instead of extruding all the nuclear contents as in suicidal NETosis, in vital NETosis, the DNA is released within vesicles, and the neutrophils phagocytic functions remain intact (51). *In vivo* imaging of neutrophils during gram-positive skin infections, have demonstrated that NET forming neutrophils can still effectively crawl despite being anuclear, and still have the ability to kill bacteria via phagocytosis (51).

Mitochondrial (mt) NETosis has also been observed, like normal NETosis it is ROS dependent. Mitochondrial nets have been formed *in vitro* in response to stimulation by GM-CSF and C5a (66). Recently mitochondrial NETosis has been demonstrated in low density neutrophils from lupus nephritis patients (67). The mitochondria formed NETs were pro-inflammatory and were able to induce production of type 1 interferons. Using MRL/*lpr (l*upus prone mice), it was shown that the *in vivo* administration of MitoTEMPO an inhibitor of mitochondrial ROS was able to reduce NETosis, and ameliorate autoimmunity in the MRL/*lpr* mice (67).

## Release of DNA From Other Leukocytes Other Than Neutrophils Is Proinflammatory

Neutrophils are not the only cells that release DNA. It has been recently described that activated CD4 T cells can release extracellular DNA traps (68). *Ex vivo* cultured Human CD4 T cells and naïve CD4+T cells from mice produced DNA threads when activated with anti-CD3/antiCD28 antibodies for 24 h. The DNA released is highly oxidized, coated with histones similar to what is see in the generation of NETs. The expelled DNA from CD4 T cells, was also detectable *in vivo* in the lymph nodes of EAE mice. The generation of these T cells is dependent on the generation of reactive oxygen species (ROS). Using the mitochondrial ROS inhibitor SkQ1 at a low dose *in vivo* reduces excess mitochondrial ROS and is protective in a model of EAE. Antigen specific (MOG_35−55_) *ex vivo* stimulation of lymph node cells from mice treated with SkQ1 had reduced immune responses and reduction in pro-inflammatory cytokines.

B cells release mitochondrial DNA in response to stimulation with CpG and non-CpG oligonucleotides (ODN) but no other known NET stimulators (69). This is independent of typical cell death such as necrosis or apoptosis. Unlike NETs this process is rapid, happening within minutes of stimulation, does not rely on ROS or NADPH. B cell expulsion of DNA is of mitochondrial origin and released in full size rather than fragments. B cell mt DNA release, acts like a DAMP and induces the production of Type 1 IFNs in co-cultures PBMCs. Inhibition of B cell mt DNA is not accomplished by inhibitors of other types of cell death, or by blocking ROS. The only treatment effective in blocking the release of B cell mt DNA was ZnCl_2_.

This data indicates when considering the inhibition of NETs, the effectiveness should be assessed also in other cells leukocytes and lymphocytes which may be contributing to inflammation through the release of ecDNA.

## NET Derived Extracellular DNA Is Pro-inflammatory

One of the major components of NETs is the backbone DNA structure. Within NETs this backbone of DNA provides the substrate that the neutrophil enzymes, proteases, and histones adhere to. Although the production of NETs helps to fight infection by containing the spread of infectious bacteria, the DNA itself can cause inflammation through activation of DNA sensors, and histone exposure (as discussed in previous section). DNA is released from dying cells through several different mechanisms including apoptosis, necrosis, necroptosis, and pyroptosis. Besides the different signaling pathways involved and pathological appearance of these types of cell deaths, the size of the DNA and quantity released during death differs also.

DNA derived from NETs can activate the immune system by acting as a danger signal. The nuclear location of DNA offers protection from exposure to the immune system under normal healthy conditions. Self-DNA which is released extracellularly during either pathogenic processes or autoimmunity is seen by the immune system as a Danger Associated Molecular Pattern (DAMP) where as non-host DNA is recognized as a Pathogen Associated Molecular Pattern (PAMPS) by Pattern Recognition Receptors (PRRs). DNA which escapes the nucleus, is detected by several different sensors. TLR9 recognition of DNA occurs within endosomes. Within the cytoplasm extra nuclear DNA is detected by either three prime repair enzymes, exonuclease 1 (TREX1), or cyclic GMP-AMP synthase (cGAS). This recognition of extra nuclear DNA activates the adapter protein Stimulator of Interferon Genes (STING). The different DNA sensors can also control which pathway of cell death may occur. For instance, extracellular DNA activation of TLR9 will induce apoptosis, activation of RIP3 will activate necrosis and NETosis, caspase 9 will trigger apoptosis, and activation of AIM2 will induce pyroptosis.

## Targeting NETs as Therapy in AAV

There are currently no alternative proven safe therapies that can replace immunosuppressive cyclophosphamide or rituximab for the treatment of AAV. Both these treatments render patients susceptible to infection (70, 71). Cyclophosphamide suppresses bone marrow production of all leukocytes and therefore immunity is unable to be restored quickly. White blood cell counts can take weeks to return to an acceptable level leaving patients vulnerable to infection, and with a worsened prognosis upon pathogen insult. While non-targeted immunosuppression can attenuate AAV, critical functions for maintaining host immunity are compromised, resulting in severe and life-threatening side effects, specifically increased susceptibility to opportunistic infections and malignancy.

### Potential Strategies to Prevent NETosis

Recent evidence suggests targeting some cell death pathways, particularly NETosis may be safer than conventional therapies. This is because emerging inhibitors of some critical NETosis specific enzymes have few off target effects. However, many of the potential NETosis inhibitors are reversible or have short half-lives. Early infection detection and rapid withdrawal would minimize the risk of increased susceptibility to infection. A summary table of inflammatory mediators released by NETs and pathways with potential therapeutic avenues can be found in Table 1 and experimental evidence demonstrating a pathogenic role of NETS in disease initiation/progression are summarized in Table 2.

**Table 1:** Inflammatory mediators released by Neutrophil Extracellular Traps and potential inhibitors.

**Molecule**	**Model of investigation**	**Inhibitor**	**References**
**Inflammatory mediators released by Neutrophil extracellular traps**			
Myeloperoxidase (MPO)	Experimental AAV, clinical MPO	AZM198	(59, 72)
Proteinase 3 (PR3)			
Neutrophil Elastase (NE)	Lung disease	Alvelestat	(73–76)
		BAY 85-8501	(77–79)
Histones	Experimental model of GN	Anti-histone antibody	(48)
DNA	Animal models of AAV, Animal model of chronic neutrophilia	DNase 1 DNase 1L3	(22, 80, 81)
mtDNA	Animal model of EAE	SkQ1	(68)
Reactive oxygen species (ROS)	Animal model of EAE	SkQ1	(68)
High Mobility Group Box 1 (HMGB1)	Animal models of sepsis, and IRI	Glycyrrhizin Gabexate mesilate HMGB1 specific ab	(82, 83)
S100A8/A9	SLE, T1D Clinical trials	Quinoline-3-carboxamide	(84, 85)
**Molecular targets in the initiation of NETS**			
Peptidyl arginine deiminase 4 (PAD4)	PTU induced AAV NET inhibition in Lupus prone MRL/lpr mice	Cl amidine Cl amidine	(86) (87)
	Experimental AAV Experimental AAV	BB-Cl amidine GSK484	(88) (88)
Gasdermin D	Human and murine neutrophils	LDC7559	(36)
RIPk1	Mouse NETs	Necrostatin (NEC)	(89)
RIPk3	Mouse model of AAV	Necrostatin (NEC1)	(22)
MLKL	Mouse model of AAV	Necorusulfonamide	(22)

**Table 2:** Experimental evidence of the pathogenic role of NETs in AAV.

**Model**	**Experimental evidence**	**References**
AAV PBMC and kidney biopsies	PR3-ANCA and MPO-ANCA stimulate NET formation *ex vivo*, glomerular NETs in AAV patient biopsies	(58)
AAV kidney biopsies	Extracellular MPO associated with glomerular NET formation, description of macrophage extracellular traps	(59)
AAV PBMCs	AAV patient neutrophils more likely to spontaneously NET than healthy controls	(62)
Animal model of AAV	NET formation can be inhibited by blocking the Necroptosis pathway	(22)
Animal model of AAV	NETs transfer neutrophil antigens in AAV	(41)
AAV PBMCs	NET formation can occur independent of ANCA, and complement pathway.	(62, 64)
AAV serum with healthy donor neutrophils	AAV NETs distinct morphology compare to SLE NETs	(65)
AAV Case Reports	NETs present in glomeruli of biopsy	(60, 61)
PTU induced animal model of AAV	Cl amidine prevented MPO-ANCA production	(86)
PTU induced Wistar-Kyoto Rat model of AAV	Pharmaceutical Immunoglobulins reduce NETs and attenuated MPO-AAV	(90)
Animal model of AAV	DNase I reduces NET formation and ameliorates AAV	(81)
Animal model of AAV	NE^−/−^ mice are NET depleted and protected from development of AAV	(91)
Animal model of AAV	PAD4^−/−^ mice have significantly less glomerular NETs and attenuated AAV	(88)
Animal model of AAV	PAD inhibitors BB-Cl amidine and GSK484 inhibit NET formation and protect attenuate development of AAV	(88)

### Peptidyl Arginase Deiminases

There are several potential candidates for targeting NETosis as therapy in autoimmune diseases, including Cl-amidine (and the second generation BB-Cl amidine), both pan PAD inhibitors, DNase I which specifically targets and clears DNA and gasdermin D inhibitors which will prevent both pyroptosis and NETosis. Multiple studies have shown a protective effect in autoimmune diseases when PAD4^−/−^ animals have been used, so it would be likely that a PAD inhibitor would have some effect in ameliorating autoimmunity in diseases in which NETs are likely to play a role such as lupus, atherosclerosis, arthritis, diabetes, and AAV (86, 87, 92–95). Although Cl-amidine and BB-Cl amidine which has a longer half-life *in vivo* has been beneficial in animal models, there are concerns that in human diseases that the inhibition of all the PADs not just PAD4 which is implicated in NET formation could have a detrimental effect in patients by increasing their susceptibility to infections. Specific inhibitors for PAD4 with no off target effects are emerging. Our laboratory has shown the use of GSK484 a specific PAD4 inhibitor and the pan inhibitor BB-CLA are protective in animal model of AAV. With a reduction in NETs, extracellular MPO and neutrophils recruitment to glomeruli. Interestingly BB-CLA is also able to induce an increase in numbers of MPO specific T regulatory cells (88).

### Deoxyribonucleases (DNase)

The deoxyribonuclease family can be divided into two broad classes based on their biochemical structure and functional properties. The DNase I family consists of DNase I, DNase 1L1, DNase 1L2, and DNase1L3. Little is known about the functions of DNase L1, and DNase 1L2. DNase L1 is produced mainly by muscle and myocardium whereas DNase 1L2 is produced in varied sites including the brain lungs, placenta and skin. All of the DNases cleave DNA, for the purposes of this review we will concentrate on DNase I and DNase 1L3 which have both been implicated in regulating ecDNA in autoimmunity.

DNase I family enzymes require bivalent magnesium and calcium ions for activation. Without magnesium DNase I is unable to efficiently cleave DNA phosphodiester bonds. DNase I is mainly produced by the pancreas, and salivary and parotid glands and released into the blood stream. G-Actin forms a complex with DNase I and inactivates it, the biological significance of this is not yet known, however this does suggest that DNase I is subjected to regulation *in vivo*. DNase I preferentially degrades double stranded DNA (dsDNA) over single stranded DNA (ssDNA). The primary role of DNase I is to degrade extracellular nuclear proteins- any disruption in this process has the potential to cause autoimmunity.

DNase1l3 is expressed highly in the lymphoid organs, and has a role in promoting plasma ecDNA homeostasis. DNase1l3 enhances fragmentation of DNA and targets nucleosomes and DNA protein complexes. DNase1l3 plays a role both in intra and extracellular degradation of DNA. It has two nuclear localization signals unlike DNase 1 which only has one in the N-terminal signal. It is proposed that this is why DNase1l3 may have a role in apoptosis where it enhances chromatin cleavage.

DNase I and DNase 1l3 both degrade NETs *in vitro* and *in vivo*, providing dual protection against excess NET formation (80). Mice genetically deficient in either DNase I or DNase 1l3 are protected against chronic neutrophilia induced by G-CSF. This indicates that in the absence of one DNase the other has the ability to compensate. However, if mice are both DNase I and DNase 1l3 deficient, mice die within 5 days. Mutations in both DNase I and DNase1l3 are associated with autoimmune diseases (96, 97). Mice deficient in DNase I and DNase 1l3 spontaneously develop SLE (98–100).

DNase I, has been shown to be safe and tolerable in a clinical trial in lupus, although not effective in treating the disease possibly due to the heterogeneity of the disease pathogenesis in Lupus, and the disease progression not reliant on immunoreactivity to DNA alone. However, valuable information on the safety and side effects can be taken from the trial, and applied to other forms of autoimmunity where extracellular DNA may play more of an injurious role (101).

Experimental evidence indicates that DNase I is therapeutic in two different models of ANCA vasculitis. Schreiber et al. demonstrated in a model of AAV that DNase I prevents ANCA induced NET formation, glomerular injury and protects mice from development of disease. MPO-ANCA activated NETs incubated with endothelial cells *in vitro* increased albumin permeability, which pre-treatment with DNase I prevented (22). Our own laboratory has shown that DNase I prevents the development of AAV, through a reduction in ecDNA released during cell death, reduced NET formation and an associated decrease in the deposition of the autoantigen MPO. DNase I is able to reduce autoimmunity to MPO with a reduced number of MPO specific lymphocytes (IL17a and IFNγ) in the draining lymph nodes and an increase in the number of MPO specific T regulatory cells, suggesting that DNase I may have an immunomodulatory role. DNase I given at the time of antigen presentation reduced the numbers of CD11c positive DCs, and activation markers MHCII, CD40, CD80, CD86, and the numbers of activated CD4+ CD69+ T cells (81).

Treatment with DNase I is not without difficulties. To achieve a therapeutic benefit in animal models of AAV, DNase I is administered intravenously twice daily to combat the short half-life (3–4 h) of DNase I in the circulation (22, 81). Recombinant DNase I is rapidly inactivated by G actin therefore the benefits of an inhaled actin resistant DNase is being currently investigated in Phase I and II clinical trials for cystic fibrosis patients (NCT02605590, NCT02722122). Other avenues to combat the limited half-life of DNase I in the circulation involve using gene delivery vectors derived from adeno-associated virus vectors (AAvec). AAvec would overcome the critical issue of the short half-life of the enzyme to provide a “one shot” therapeutic for SLE and ANCA-vasculitis through continuous delivery of DNase I. AAvec technology has already been used successfully to treat blindness, clotting disorders, and neuromuscular disease in humans (102). Over expression of both DNase I and DNase Il3 protein with pLIVE plasmids enables the liver to produce long lasting over expression of both proteins in mice. In a model of sepsis in DNase I^−/−^ and DNase 1l3^−/−^ mice, plasmid expression of DNase I and DNase 1l3 restores endogenous circulating levels of both types of DNase and prevents vascular occlusion from NETosis in a model of chronic neutrophilia (80).

DNase I coated melanin-like nanospheres have been used successfully to control NET dysregulation in a model of sepsis (103). To circumvent the relative short half-life of DNase I in the circulation bare bioinspired melanin-like nanospheres (bMNSs) are coated with polydopamine which immobilizes DNase I on the surface of the bMNSs. The DNase I bMNSs retain biological activity for 36 h. Intravenous injection of DNase I bMNSs significantly reduced NETosis, extracellular DNA levels, neutrophil counts, reduced MPO and NE activity, and proinflammatory cytokines in a murine model of sepsis. Mice given DNase I bMNSs had improved survival rates compared to mice given endogenous DNase I.

In addition to clearing NET DNA DNase I can clear DNA from other types of cell death where DNA is released into the extracellular environment such as apoptosis, necrosis, and pyroptosis. One limitation of DNase I is that it only dismantles formed NETs. Some NET remnants (histones and proteases) are left behind in vessel walls (104). DNase I as adjunctive therapy may allow reduction in the dose of currently used effective drugs particularly steroids, where high dose related side effects are common.

### Neutrophil Elastase Inhibitors

The role of neutrophil elastase (NE) as a major constituent of NETs is well-accepted however the role of NE in instigating NETosis is controversial. NE was first shown in 2010 to work synergistically with MPO to break down the nuclear envelope and help cleave histones as discussed in a previous section (29). These findings were confounded when it was found that NE^−/−^ mice were able to form NETs in a model of deep vein thrombosis, putting into dispute the role of NE in forming NETs (105). Mice with a genetic deficiency in NE only had a modest reduction in NET formation compared to WT controls when ionomycin was used to stimulate NET formation. The conflict in these results suggests that NE plays a different role in instigating NETosis dependent on the stimulus. It is also possible in the absence of NE another protease takes over the role of regulation of NETosis. Recently it has been shown that GSDMD and NE work synergistically to break down the nuclear envelope and the outer cell membrane during NETosis highlighting again the importance of NE in NETosis and its relevance as a therapeutic target, both in instigating NETs and neutralizing once it has been exteriorized thorough NETosis (36).

Two promising candidates for neutrophil elastase inhibitors are Alvelestat and BAY 85-8501, both have been shown to be safe and tolerable in clinical trials for the treatment of airway diseases so is poised for clinical translation to target NETosis in AAV.

Alvelestat (AZD9668), is a 3rd generation neutrophil elastase inhibitor which is reversible and highly selective for Neutrophil Elastase and to a lesser extent PR3 (77). It has been utilized in Phase II clinical trials for the treatment of air way diseases at a dose of 60 mg b.i.d (twice daily) including cystic fibrosis (73), chronic obstructive pulmonary disease (74), and bronchiectasis (75). Alvelestat has been effective in inhibiting NETs in a model of acute lung injury/acute respiratory distress syndrome in mice and significantly reducing the inflammatory response (76).

BAY 85-8501 is a 5th generation neutrophil elastase inhibitor with potency similar to that of endogenous antiproteases, it has improved metabolic stability over the previous generation of inhibitors with an improved half-life. Phase I clinical trials with healthy males showed that a dose of up to 1 mg is well-tolerated with no evidence of adverse reactions (78). Phase II clinical trials have shown Bay 85-8501 to be safe for the treatment of non-cystic fibrosis bronchiectasis (79), and bronchiectasis (75). BAY 85-8501 reduces human primary neutrophil NET formation *in vitro* when stimulated with PMA (36). Based on the results of the safety profile of BAY 85-8501 and the *in vitro* capability of inhibiting NET formation, it is a promising candidate to block NET formation in AAV.

Inhibition of NE raises the possibility that host defense will be compromised. This is minimized with the use of the most recent generation of NE inhibitors as the inhibitors are reversible and have been shown to be dose dependent in *ex vivo* blood zymosan assays of neutrophils to determine the phagocytosis capability after use of the NE inhibitors (77). Of clinical relevance is preliminary data that they can inhibit NE activity to a level that has clinical benefit (between 50 and 90%) but does not reduce the innate capacity of neutrophils to engulf and kill bacteria (77).

Our laboratory has shown that neutrophil elastase knock mice (*Elane*^−/−^*)* are protected from the development of murine MPO-AAV with a significant reduction in albuminuria/creatine ratio, glomerular segmental necrosis, glomerular NETs and associated MPO deposition, recruitment of glomerular CD4+ T cells, macrophages and neutrophils (91). Both Alvelestat and BAY 85-8501 have been used to inhibit NETs in a model of MPO-AAV with favorable results. Daily administration of the NE inhibitors via oral gavage reduced glomerular injury, recruitment of glomerular CD4 T cells, CD8 T cells, macrophages and neutrophils. Glomerular NET accumulation and MPO deposition was significantly reduced. ANCA was significantly reduced in the BAY 85-8501 treated mice but not in the Alvelestat treated mice.

Collectively these experimental results demonstrate that NE inhibition is protective in experimental MPO-AAV. Favorable results from clinical trials in terms of safety and efficacy are encouraging for the investigation of clinical translation of Alvelestat and BAY 85-8501 for the treatment of MPO-AAV (106).

## Concluding Remarks

Current treatment for AAV is suboptimal with unavoidable adverse effects. A targeted more specific treatment is required. NETs are a common feature within glomeruli of kidneys from MPO-AAV patients. The key autoantigens in AAV MPO and PR3 are released during NET formation along with other potential injurious molecules that cause direct injury to glomerular endothelial cells. Therapeutically targeting NETs will prevent the release of these autoantigens, reducing glomerular damage, with the potential to ameliorate the progression of AAV.

## Author Contributions

All authors listed have made a substantial, direct and intellectual contribution to the work, and approved it for publication.

## Conflict of Interest

The authors declare that the research was conducted in the absence of any commercial or financial relationships that could be construed as a potential conflict of interest.

## Abbreviations

AAV: Anti Neutrophil Cytoplasmic Antibody
ANCA: Anti Neutrophil Cytoplasmic Antibody Associated Vasculitis
Cl: Chlorine
DAMPS: Danger Associated Molecular Associated Patterns
DNA: Deoxyribonuclease
dsDNA: Double Stranded Deoxyribonuclease
DTH: Delayed Type Hypersensitivity
EPA: Eosinophilic Granulomatosis with Polyangiitis
GN: Glomerulonephritis
GPA: Granulamotisis with Polyangiitis
H2O2: Hydrogen Peroxidise
HMGB1: High Mobility Group Box 1
IgG: Immunoglobulin
IRI: Ischemia Reperfusion Injury
LPS: Liposaccharide
MLKL: Mixed Linked Kinase Domain-like
MPA: Microscopic Polyangiitis
MPO: Myeloperoxidase
MPO-AAV: Myeloperoxidase ANCA associated vasculitis
mtDNA: Mitochondrial Deoxyribonuclease
NADPH: Nicotinamide Adenine Dinucleotide Phosphate
NE: Neutrophil Elastase
Nec 1: Necrostatin 1
NETs: Neutrophil Extracellular Traps
PAD4: Peptidyl Arginine Deiminase
PMNCs: Polymorphonuclear cells
PR3: Proteinase 3
PTU: Propylthiouracil
RIPK1: Receptor interacting Protein Kinases-3
ROS: Reactive Oxygen Species
RPCGN: Rapidly Progressive Crescentic Glomerulonephritis
SLE: Systemic Lupus Erythematosus.

## References

[1] Arimura Y Muso E Fujimoto S Hasegawa M Kaname S Usui J. Evidence-based clinical practice guidelines for rapidly progressive glomerulonephritis 2014. Clin Exp Nephrol. (2016) 20:322–41. 10.1007/s10157-015-1218-8 27099135 PMC4891375

[2] Greenhall GH Salama AD. What is new in the management of rapidly progressive glomerulonephritis? Clin Kidney J. (2015) 8:143–50. 10.1093/ckj/sfv008 25815169 PMC4370308

[3] Ball GV. The history of ANCA-associated vasculitis. Rheum Dis Clin North Am. (2010) 36:439–46. 10.1016/j.rdc.2010.05.004 20688242

[4] Hamour S Salama AD Pusey CD. Management of ANCA-associated vasculitis: current trends and future prospects. Ther Clin Risk Manag. (2010) 6:253–64. 10.2147/TCRM.S6112 20596502 PMC2893757

[5] Jayne D. Review article: Progress of treatment in ANCA-associated vasculitis. Nephrology. (2009) 14:42–8. 10.1111/j.1440-1797.2009.01101.x 19335843

[6] Morgan MD Harper L Williams J Savage C. Anti-neutrophil cytoplasm-associated glomerulonephritis. J Am Soc Nephrol. (2006) 17:1224–34. 10.1681/ASN.2005080882 16624931

[7] Hiemstra TF Walsh M Mahr A Savage CO de Groot K Harper L. Mycophenolate mofetil vs azathioprine for remission maintenance in antineutrophil cytoplasmic antibody-associated vasculitis: a randomized controlled trial. JAMA. (2010) 304:2381–8. 10.1001/jama.2010.1658 21060104

[8] Xiao H Dairaghi DJ Powers JP Ertl LS Baumgart T Wang Y. C5a receptor (CD88) blockade protects against MPO-ANCA GN. J Am Soc Nephrol. (2014) 25:225–31. 10.1681/ASN.2013020143 24179165 PMC3904560

[9] Falk RJ Terrell RS Charles LA Jennette JC. Anti-neutrophil cytoplasmic autoantibodies induce neutrophils to degranulate and produce oxygen radicals *in vitro*. Proc Natl Acad Sci USA. (1990) 87:4115–9. 10.1073/pnas.87.11.4115 2161532 PMC54058

[10] Brouwer E Huitema MG Mulder AH Heeringa P van Goor H Tervaert JW. Neutrophil activation *in vitro* and *in vivo* in Wegener's granulomatosis. Kidney Int. (1994) 45:1120–31. 10.1038/ki.1994.149 8007582

[11] Xiao H Heeringa P Hu P Liu Z Zhao M Aratani Y. Antineutrophil cytoplasmic autoantibodies specific for myeloperoxidase cause glomerulonephritis and vasculitis in mice. J Clin Invest. (2002) 110:955–63. 10.1172/JCI0215918 12370273 PMC151154

[12] Xiao H Heeringa P Liu Z Huugen D Hu P Maeda N. The role of neutrophils in the induction of glomerulonephritis by anti-myeloperoxidase antibodies. Am J Pathol. (2005) 167:39–45. 10.1016/S0002-9440(10)62951-3 15972950 PMC1603451

[13] Porges AJ Redecha PB Kimberly WT Csernok E Gross WL Kimberly RP. Anti-neutrophil cytoplasmic antibodies engage and activate human neutrophils via Fc gamma RIIa. J Immunol. (1994) 153:1271–80. 8027554

[14] Kocher M Edberg JC Fleit HB Kimberly RP. Antineutrophil cytoplasmic antibodies preferentially engage Fc gammaRIIIb on human neutrophils. J Immunol. (1998) 161:6909–14. 9862724

[15] Kettritz R Jennette JC Falk RJ. Crosslinking of ANCA-antigens stimulates superoxide release by human neutrophils. J Am Soc Nephrol. (1997) 8:386–94. 9071707 10.1681/ASN.V83386

[16] Dick J Gan PY Ford SL Odobasic D Alikhan MA Loosen SH. C5a receptor 1 promotes autoimmunity, neutrophil dysfunction and injury in experimental anti-myeloperoxidase glomerulonephritis. Kidney Int. (2018) 93:615–25. 10.1016/j.kint.2017.09.018 29241626

[17] Xiao H Schreiber A Heeringa P Falk RJ Jennette JC. Alternative complement pathway in the pathogenesis of disease mediated by anti-neutrophil cytoplasmic autoantibodies. Am J Pathol. (2007) 170:52–64. 10.2353/ajpath.2007.060573 17200182 PMC1762697

[18] Schreiber A Xiao H Jennette JC Schneider W Luft FC Kettritz R. C5a receptor mediates neutrophil activation and ANCA-induced glomerulonephritis. J Am Soc Nephrol. (2009) 20:289–98. 10.1681/ASN.2008050497 19073822 PMC2637051

[19] Jayne DRW Bruchfeld AN Harper L Schaier M Venning MC Hamilton P. Randomized trial of C5a receptor inhibitor avacopan in ANCA-associated vasculitis. J Am Soc Nephrol. (2017) 28:2756–67. 10.1681/ASN.2016111179 28400446 PMC5576933

[20] Fiers W Beyaert R Declercq W Vandenabeele P. More than one way to die: apoptosis, necrosis and reactive oxygen damage. Oncogene. (1999) 18:7719–30. 10.1038/sj.onc.1203249 10618712

[21] Pasparakis M Vandenabeele P. Necroptosis and its role in inflammation. Nature. (2015) 517:311–20. 10.1038/nature14191 25592536

[22] Schreiber A Rousselle A Becker JU von Massenhausen A Linkermann A Kettritz R. Necroptosis controls NET generation and mediates complement activation, endothelial damage, and autoimmune vasculitis. Proc Natl Acad Sci USA. (2017) 114:E9618–25. 10.1073/pnas.1708247114 29078325 PMC5692554

[23] Brinkmann V Reichard U Goosmann C Fauler B Uhlemann Y Weiss DS. Neutrophil extracellular traps kill bacteria. Science. (2004) 303:1532–5. 10.1126/science.1092385 15001782

[24] Fuchs TA Abed U Goosmann C Hurwitz R Schulze I Wahn V. Novel cell death program leads to neutrophil extracellular traps. J Cell Biol. (2007) 176:231–41. 10.1083/jcb.200606027 17210947 PMC2063942

[25] Lundberg K Nijenhuis S Vossenaar ER Palmblad K van Venrooij WJ Klareskog L. Citrullinated proteins have increased immunogenicity and arthritogenicity and their presence in arthritic joints correlates with disease severity. Arthritis Res Ther. (2005) 7:R458–67. 10.1186/ar1697 15899032 PMC1174941

[26] Neeli I Khan SN Radic M. Histone deimination as a response to inflammatory stimuli in neutrophils. J Immunol. (2008) 180:1895–902. 10.4049/jimmunol.180.3.1895 18209087

[27] Wang Y Li M Stadler S Correll S Li P Wang D. Histone hypercitrullination mediates chromatin decondensation and neutrophil extracellular trap formation. J Cell Biol. (2009) 184:205–13. 10.1083/jcb.200806072 19153223 PMC2654299

[28] Li P Li M Lindberg MR Kennett MJ Xiong N Wang Y. PAD4 is essential for antibacterial innate immunity mediated by neutrophil extracellular traps. J Exp Med. (2010) 207:1853–62. 10.1084/jem.20100239 20733033 PMC2931169

[29] Papayannopoulos V Metzler KD Hakkim A Zychlinsky A. Neutrophil elastase and myeloperoxidase regulate the formation of neutrophil extracellular traps. J Cell Biol. (2010) 191:677–91. 10.1083/jcb.201006052 20974816 PMC3003309

[30] Metzler KD Goosmann C Lubojemska A Zychlinsky A Papayannopoulos V. A myeloperoxidase-containing complex regulates neutrophil elastase release and actin dynamics during NETosis. Cell Rep. (2014) 8:883–96. 10.1016/j.celrep.2014.06.044 25066128 PMC4471680

[31] Klebanoff SJ Kettle AJ Rosen H Winterbourn CC Nauseef WM. Myeloperoxidase: a front-line defender against phagocytosed microorganisms. J Leukoc Biol. (2013) 93:185–98. 10.1189/jlb.0712349 23066164 PMC3545676

[32] Bjornsdottir H Welin A Michaelsson E Osla V Berg S Christenson K. Neutrophil NET formation is regulated from the inside by myeloperoxidase-processed reactive oxygen species. Free Radic Biol Med. (2015) 89:1024–35. 10.1016/j.freeradbiomed.2015.10.398 26459032

[33] Rochael NC Guimaraes-Costa AB Nascimento MT DeSouza-Vieira TS Oliveira MP Garcia e Souza LF. Classical ROS-dependent and early/rapid ROS-independent release of Neutrophil Extracellular Traps triggered by Leishmania parasites. Sci Rep. (2015) 5:18302. 10.1038/srep18302 26673780 PMC4682131

[34] Parker H Dragunow M Hampton MB Kettle AJ Winterbourn CC. Requirements for NADPH oxidase and myeloperoxidase in neutrophil extracellular trap formation differ depending on the stimulus. J Leukoc Biol. (2012) 92:841–9. 10.1189/jlb.1211601 22802447

[35] Bergsbaken T Fink SL Cookson BT. Pyroptosis: host cell death and inflammation. Nat Rev Microbiol. (2009) 7:99–109. 10.1038/nrmicro2070 19148178 PMC2910423

[36] Sollberger G Choidas A Burn GL Habenberger P Di Lucrezia R Kordes S. Gasdermin D plays a vital role in the generation of neutrophil extracellular traps. Sci Immunol. (2018) 3:1–12. 10.1126/sciimmunol.aar6689 30143555

[37] Chen KW Monteleone M Boucher D Sollberger G Ramnath D Condon ND. Noncanonical inflammasome signaling elicits gasdermin D-dependent neutrophil extracellular traps. Sci Immunol. (2018) 3:1–11. 10.1126/sciimmunol.aar6676 30143554

[38] Hakkim A Fuchs TA Martinez NE Hess S Prinz H Zychlinsky A. Activation of the Raf-MEK-ERK pathway is required for neutrophil extracellular trap formation. Nat Chem Biol. (2011) 7:75–7. 10.1038/nchembio.496 21170021

[39] Desai J Kumar SV Mulay SR Konrad L Romoli S Schauer C. PMA and crystal-induced neutrophil extracellular trap formation involves RIPK1-RIPK3-MLKL signaling. Eur J Immunol. (2016) 46:223–9. 10.1002/eji.201545605 26531064

[40] Amini P Stojkov D Wang X Wicki S Kaufmann T Wong WW. NET formation can occur independently of RIPK3 and MLKL signaling. Eur J Immunol. (2016) 46:178–84. 10.1002/eji.201545615 26549703 PMC4738457

[41] Sangaletti S Tripodo C Chiodoni C Guarnotta C Cappetti B Casalini P. Neutrophil extracellular traps mediate transfer of cytoplasmic neutrophil antigens to myeloid dendritic cells toward ANCA induction and associated autoimmunity. Blood. (2012) 120:3007–18. 10.1182/blood-2012-03-416156 22932797

[42] Marin-Gallen S Clemente-Casares X Planas R Pujol-Autonell I Carrascal J Carrillo J. Dendritic cells pulsed with antigen-specific apoptotic bodies prevent experimental type 1 diabetes. Clin Exp Immunol. (2010) 160:207–14. 10.1111/j.1365-2249.2009.04082.x 20030670 PMC2857943

[43] Xu J Zhang X Pelayo R Monestier M Ammollo CT Semeraro F. Extracellular histones are major mediators of death in sepsis. Nat Med. (2009) 15:1318–21. 10.1038/nm.2053 19855397 PMC2783754

[44] Abrams ST Zhang N Manson J Liu T Dart C Baluwa F. Circulating histones are mediators of trauma-associated lung injury. Am J Respir Crit Care Med. (2013) 187:160–9. 10.1164/rccm.201206-1037OC 23220920 PMC3570656

[45] Ammollo CT Semeraro F Xu J Esmon NL Esmon CT. Extracellular histones increase plasma thrombin generation by impairing thrombomodulin-dependent protein C activation. J Thromb Haemost. (2011) 9:1795–803. 10.1111/j.1538-7836.2011.04422.x 21711444

[46] Allam R Scherbaum CR Darisipudi MN Mulay SR Hagele H Lichtnekert J. Histones from dying renal cells aggravate kidney injury via TLR2 and TLR4. J Am Soc Nephrol. (2012) 23:1375–88. 10.1681/ASN.2011111077 22677551 PMC3402284

[47] Huang H Chen HW Evankovich J Yan W Rosborough BR Nace GW. Histones activate the NLRP3 inflammasome in Kupffer cells during sterile inflammatory liver injury. J Immunol. (2013) 191:2665–79. 10.4049/jimmunol.1202733 23904166 PMC3777242

[48] Kumar SV Kulkarni OP Mulay SR Darisipudi MN Romoli S Thomasova D. Neutrophil extracellular trap-related extracellular histones cause vascular necrosis in severe GN. J Am Soc Nephrol. (2015) 26:2399–413. 10.1681/ASN.2014070673 25644111 PMC4587690

[49] Tillack K Breiden P Martin R Sospedra M. T lymphocyte priming by neutrophil extracellular traps links innate and adaptive immune responses. J Immunol. (2012) 188:3150–9. 10.4049/jimmunol.1103414 22351936

[50] Warnatsch A Ioannou M Wang Q Papayannopoulos V. Inflammation. Neutrophil extracellular traps license macrophages for cytokine production in atherosclerosis. Science. (2015) 349:316–20. 10.1126/science.aaa8064 26185250 PMC4854322

[51] Yipp BG Petri B Salina D Jenne CN Scott BN Zbytnuik LD. Infection-induced NETosis is a dynamic process involving neutrophil multitasking *in vivo*. Nat Med. (2012) 18:1386–93. 10.1038/nm.2847 22922410 PMC4529131

[52] Guglietta S Chiavelli A Zagato E Krieg C Gandini S Ravenda PS. Coagulation induced by C3aR-dependent NETosis drives protumorigenic neutrophils during small intestinal tumorigenesis. Nat Commun. (2016) 7:11037. 10.1038/ncomms11037 26996437 PMC4802169

[53] Yuen J Pluthero FG Douda DN Riedl M Cherry A Ulanova M. NETosing neutrophils activate complement both on their own NETs and bacteria via alternative and non-alternative pathways. Front Immunol. (2016) 7:137. 10.3389/fimmu.2016.00137 27148258 PMC4831636

[54] Palmer LJ Damgaard C Holmstrup P Nielsen CH. Influence of complement on neutrophil extracellular trap release induced by bacteria. J Periodontal Res. (2016) 51:70–6. 10.1111/jre.12284 25900429

[55] Huang YM Wang H Wang C Chen M Zhao MH. Promotion of hypercoagulability in antineutrophil cytoplasmic antibody-associated vasculitis by C5a-induced tissue factor-expressing microparticles and neutrophil extracellular traps. Arthritis Rheumatol. (2015) 67:2780–90. 10.1002/art.39239 26097236

[56] Vogt W. Complement activation by myeloperoxidase products released from stimulated human polymorphonuclear leukocytes. Immunobiology. (1996) 195:334–46. 10.1016/S0171-2985(96)80050-7 8877407

[57] Venge P Olsson I. Cationic proteins of human granulocytes. VI. Effects on the complement system and mediation of chemotactic activity. J Immunol. (1975) 115:1505–8. 1184964

[58] Kessenbrock K Krumbholz M Schonermarck U Back W Gross WL Werb Z. Netting neutrophils in autoimmune small-vessel vasculitis. Nat Med. (2009) 15:623–5. 10.1038/nm.1959 19448636 PMC2760083

[59] O'Sullivan KM Lo CY Summers SA Elgass KD McMillan PJ Longano A. Renal participation of myeloperoxidase in antineutrophil cytoplasmic antibody (ANCA)-associated glomerulonephritis. Kidney Int. (2015) 88:1030–46. 10.1038/ki.2015.202 26176828

[60] Yoshida M Sasaki M Sugisaki K Yamaguchi Y Yamada M. Neutrophil extracellular trap components in fibrinoid necrosis of the kidney with myeloperoxidase-ANCA-associated vasculitis. Clin Kidney J. (2013) 6:308–12. 10.1093/ckj/sft048 26064491 PMC4400491

[61] Nakazawa D Tomaru U Yamamoto C Jodo S Ishizu A. Abundant neutrophil extracellular traps in thrombus of patient with microscopic polyangiitis. Front Immunol. (2012) 3:333. 10.3389/fimmu.2012.00333 23162551 PMC3495275

[62] Soderberg D Kurz T Motamedi A Hellmark T Eriksson P Segelmark M. Increased levels of neutrophil extracellular trap remnants in the circulation of patients with small vessel vasculitis, but an inverse correlation to anti-neutrophil cytoplasmic antibodies during remission. Rheumatology. (2015) 54:2085–94. 10.1093/rheumatology/kev217 26170375

[63] Wang H Sha LL Ma TT Zhang LX Chen M Zhao MH. Circulating level of neutrophil extracellular traps is not a useful biomarker for assessing disease activity in antineutrophil cytoplasmic antibody-associated vasculitis. PLoS ONE. (2016) 11:e0148197. 10.1371/journal.pone.0148197 26840412 PMC4739550

[64] Kraaij T Kamerling SWA van Dam LS Bakker JA Bajema IM Page T. Excessive neutrophil extracellular trap formation in ANCA-associated vasculitis is independent of ANCA. Kidney Int. (2018) 94:139–49. 10.1016/j.kint.2018.01.013 29606398

[65] van Dam LS Kraaij T Kamerling SWA Bakker JA Scherer UH Rabelink TJ. Intrinsically distinct role of neutrophil extracellular trap formation in antineutrophil cytoplasmic antibody-associated vasculitis compared to systemic lupus erythematosus. Arthritis Rheumatol. (2019) 71:2047–58. 10.1002/art.41047 31313503 PMC7384043

[66] Yousefi S Mihalache C Kozlowski E Schmid I Simon HU. Viable neutrophils release mitochondrial DNA to form neutrophil extracellular traps. Cell Death Differ. (2009) 16:1438–44. 10.1038/cdd.2009.96 19609275

[67] Lood C Blanco LP Purmalek MM Carmona-Rivera C De Ravin SS Smith CK. Neutrophil extracellular traps enriched in oxidized mitochondrial DNA are interferogenic and contribute to lupus-like disease. Nat Med. (2016) 22:146–53. 10.1038/nm.4027 26779811 PMC4742415

[68] Costanza M Poliani PL Portararo P Cappetti B Musio S Pagani F. DNA threads released by activated CD4(+) T lymphocytes provide autocrine costimulation. Proc Natl Acad Sci USA. (2019) 116:8985–94. 10.1073/pnas.1822013116 30988194 PMC6500139

[69] Ingelsson B Soderberg D Strid T Soderberg A Bergh AC Loitto V. Lymphocytes eject interferogenic mitochondrial DNA webs in response to CpG and non-CpG oligodeoxynucleotides of class C. Proc Natl Acad Sci USA. (2018) 115:E478–87. 10.1073/pnas.1711950115 29295921 PMC5776968

[70] McAdoo SP Medjeral-Thomas N Gopaluni S Tanna A Mansfield N Galliford J. Long-term follow-up of a combined rituximab and cyclophosphamide regimen in renal anti-neutrophil cytoplasm antibody-associated vasculitis. Nephrol Dial Transplant. (2018) 33:899. 10.1093/ndt/gfy075 29617842 PMC7191881

[71] Jones RB Tervaert JW Hauser T Luqmani R Morgan MD Peh CA. Rituximab versus cyclophosphamide in ANCA-associated renal vasculitis. N Engl J Med. (2010) 363:211–20. 10.1056/NEJMoa0909169 20647198

[72] Antonelou M Michaelsson E Evans RDR Wang CJ Henderson SR Walker LSK. Therapeutic myeloperoxidase inhibition attenuates neutrophil activation, ANCA-mediated endothelial damage, and crescentic GN. J Am Soc Nephrol. (2020) 31:350–64. 10.1681/ASN.2019060618 31879336 PMC7003306

[73] Elborn JS Perrett J Forsman-Semb K Marks-Konczalik J Gunawardena K Entwistle N. Efficacy, safety and effect on biomarkers of AZD9668 in cystic fibrosis. Eur Respir J. (2012) 40:969–76. 10.1183/09031936.00194611 22267768

[74] Kuna P Jenkins M O'Brien CD Fahy WA. AZD9668, a neutrophil elastase inhibitor, plus ongoing budesonide/formoterol in patients with COPD. Respir Med. (2012) 106:531–9. 10.1016/j.rmed.2011.10.020 22197578

[75] Stockley R De Soyza A Gunawardena K Perrett J Forsman-Semb K Entwistle N. Phase II study of a neutrophil elastase inhibitor (AZD9668) in patients with bronchiectasis. Respir Med. (2013) 107:524–33. 10.1016/j.rmed.2012.12.009 23433769

[76] Li H Zhou X Tan H Hu Y Zhang L Liu S. Neutrophil extracellular traps contribute to the pathogenesis of acid-aspiration-induced ALI/ARDS. Oncotarget. (2018) 9:1772–84. 10.18632/oncotarget.22744 29416730 PMC5788598

[77] von Nussbaum F Li VM. Neutrophil elastase inhibitors for the treatment of (cardio)pulmonary diseases: Into clinical testing with pre-adaptive pharmacophores. Bioorg Med Chem Lett. (2015) 25:4370–81. 10.1016/j.bmcl.2015.08.049 26358162

[78] Nagelschmitz J KD Von Nussbaum F Delbeck M Lusting K Bandel T Watz H. The novel elastase inhibitor BAY 85-8501 provides a new approach in the treatment of pulmonary diseases. Eur Respirat J. (2014) 44:3416.

[79] Watz H Nagelschmitz J Kirsten A Pedersen F van der Mey D Schwers S. Safety and efficacy of the human neutrophil elastase inhibitor BAY 85-8501 for the treatment of non-cystic fibrosis bronchiectasis: a randomized controlled trial. Pulm Pharmacol Ther. (2019) 56:86–93. 10.1016/j.pupt.2019.03.009 30917927

[80] Jimenez-Alcazar M Rangaswamy C Panda R Bitterling J Simsek YJ Long AT. Host DNases prevent vascular occlusion by neutrophil extracellular traps. Science. (2017) 358:1202–6. 10.1126/science.aam8897 29191910

[81] O'Sullivan KM GP Kitching AR Holdsworth SR. Deoxyribonuclease I reduces glomerular injury and modulates antimyeloperoxidase autoimmunity in experimental anti myeloperoxidase glomerulonephritis. Rheumatology. (2017) 56:WS6–3. 10.1093/rheumatology/kex120

[82] Gong G Xiang L Yuan L Hu L Wu W Cai L. Protective effect of glycyrrhizin, a direct HMGB1 inhibitor, on focal cerebral ischemia/reperfusion-induced inflammation, oxidative stress, and apoptosis in rats. PLoS ONE. (2014) 9:e89450. 10.1371/journal.pone.0089450 24594628 PMC3942385

[83] Musumeci D Roviello GN Montesarchio D. An overview on HMGB1 inhibitors as potential therapeutic agents in HMGB1-related pathologies. Pharmacol Ther. (2014) 141:347–57. 10.1016/j.pharmthera.2013.11.001 24220159

[84] Coutant R Landais P Rosilio M Johnsen C Lahlou N Chatelain P. Low dose linomide in Type I juvenile diabetes of recent onset: a randomised placebo-controlled double blind trial. Diabetologia. (1998) 41:1040–6. 10.1007/s001250051028 9754822

[85] Bengtsson AA Sturfelt G Lood C Ronnblom L van Vollenhoven RF Axelsson B. Pharmacokinetics, tolerability, and preliminary efficacy of paquinimod (ABR-215757), a new quinoline-3-carboxamide derivative: studies in lupus-prone mice and a multicenter, randomized, double-blind, placebo-controlled, repeat-dose, dose-ranging study in patients with systemic lupus erythematosus. Arthritis Rheum. (2012) 64:1579–88. 10.1002/art.33493 22131101

[86] Kusunoki Y Nakazawa D Shida H Hattanda F Miyoshi A Masuda S. Peptidylarginine deiminase inhibitor suppresses neutrophil extracellular trap formation and MPO-ANCA production. Front Immunol. (2016) 7:227. 10.3389/fimmu.2016.00227 27375623 PMC4896908

[87] Knight JS Subramanian V O'Dell AA Yalavarthi S Zhao W Smith CK. Peptidylarginine deiminase inhibition disrupts NET formation and protects against kidney, skin and vascular disease in lupus-prone MRL/lpr mice. Ann Rheum Dis. (2015) 74:2199–206. 10.1136/annrheumdis-2014-205365 25104775 PMC4320672

[88] O'Sullivan KM GP Kitching AR Holdsworth SR. Inhibition of peptidylarginine deiminase 4 limits neutrophil extracellular trap formation and inflammation in experimental anti MPO-ANCA glomerulonephritis. Rheumatology. (2019) 58:90–1. 10.1093/rheumatology/kez061.024

[89] D'Cruz AA Speir M Bliss-Moreau M Dietrich S Wang S Chen AA. The pseudokinase MLKL activates PAD4-dependent NET formation in necroptotic neutrophils. Sci Signal. (2018) 11:1–11. 10.1126/scisignal.aao1716 30181240 PMC6301070

[90] Uozumi R Iguchi R Masuda S Nishibata Y Nakazawa D Tomaru U. Pharmaceutical immunoglobulins reduce neutrophil extracellular trap formation and ameliorate the development of MPO-ANCA-associated vasculitis. Mod Rheumatol. (2020) 30:544–50. 10.1080/14397595.2019.1602292 30932727

[91] O'Sullivan KM GP Kitching AR Holdsworth SR. Neutrophil elastase-deficient mice are protected from experimental myeloperoxidase anti-neutrophil cytoplasmic antibody vasculitis. J Am Soc Nephrol. (2019) 30:912.

[92] Knight JS Luo W O'Dell AA Yalavarthi S Zhao W Subramanian V. Peptidylarginine deiminase inhibition reduces vascular damage and modulates innate immune responses in murine models of atherosclerosis. Circ Res. (2014) 114:947–56. 10.1161/CIRCRESAHA.114.303312 24425713 PMC4185401

[93] Ghari F Quirke AM Munro S Kawalkowska J Picaud S McGouran J. Citrullination-acetylation interplay guides E2F-1 activity during the inflammatory response. Sci Adv. (2016) 2:e1501257. 10.1126/sciadv.1501257 26989780 PMC4788482

[94] Kawalkowska J Quirke AM Ghari F Davis S Subramanian V Thompson PR. Abrogation of collagen-induced arthritis by a peptidyl arginine deiminase inhibitor is associated with modulation of T cell-mediated immune responses. Sci Rep. (2016) 6:26430. 10.1038/srep26430 27210478 PMC4876390

[95] Wong SL Demers M Martinod K Gallant M Wang Y Goldfine AB. Diabetes primes neutrophils to undergo NETosis, which impairs wound healing. Nat Med. (2015) 21:815–9. 10.1038/nm.3887 26076037 PMC4631120

[96] Al-Mayouf SM Sunker A Abdwani R Abrawi SA Almurshedi F Alhashmi N. Loss-of-function variant in DNASE1L3 causes a familial form of systemic lupus erythematosus. Nat Genet. (2011) 43:1186–8. 10.1038/ng.975 22019780

[97] Yasutomo K Horiuchi T Kagami S Tsukamoto H Hashimura C Urushihara M. Mutation of DNASE1 in people with systemic lupus erythematosus. Nat Genet. (2001) 28:313–4. 10.1038/91070 11479590

[98] Wilber A O'Connor TP Lu ML Karimi A Schneider MC. Dnase1l3 deficiency in lupus-prone MRL and NZB/W F1 mice. Clin Exp Immunol. (2003) 134:46–52. 10.1046/j.1365-2249.2003.02267.x 12974753 PMC1808827

[99] Serpas L Chan RWY Jiang P Ni M Sun K Rashidfarrokhi A. Dnase1l3 deletion causes aberrations in length and end-motif frequencies in plasma DNA. Proc Natl Acad Sci USA. (2019) 116:641–9. 10.1073/pnas.1815031116 30593563 PMC6329986

[100] Sisirak V Sally B D'Agati V Martinez-Ortiz W Ozcakar ZB David J. Digestion of chromatin in apoptotic cell microparticles prevents autoimmunity. Cell. (2016) 166:88–101. 10.1016/j.cell.2016.05.034 27293190 PMC5030815

[101] Davis JC Jr Manzi S Yarboro C Rairie J McInnes I. Recombinant human Dnase I (rhDNase) in patients with lupus nephritis. Lupus. (1999) 8:68–76. 10.1191/096120399678847380 10025601

[102] Dunbar CE High KA Joung JK Kohn DB Ozawa K Sadelain M. Gene therapy comes of age. Science. (2018) 359(6372). 10.1126/science.aan4672 29326244

[103] Park HH Park W Lee YY Kim H Seo HS Choi DW. Bioinspired DNase-I-coated melanin-like nanospheres for modulation of infection-associated NETosis dysregulation. Adv Sci. (2020) 7:2001940. 10.1002/advs.202001940 33173718 PMC7645930

[104] Kolaczkowska E Jenne CN Surewaard BG Thanabalasuriar A Lee WY Sanz MJ. Molecular mechanisms of NET formation and degradation revealed by intravital imaging in the liver vasculature. Nat Commun. (2015) 6:6673. 10.1038/ncomms7673 25809117 PMC4389265

[105] Martinod K Witsch T Farley K Gallant M Remold-O'Donnell E Wagner DD. Neutrophil elastase-deficient mice form neutrophil extracellular traps in an experimental model of deep vein thrombosis. J Thromb Haemost. (2016) 14:551–8. 10.1111/jth.13239 26712312 PMC4785059

[106] O'Sullivan KM GP Kitching AR Holdsworth SR (editor). Neutrophil elastase inhibition attenuates renal inflammation in experimental MPO-ANCA vasculitis. Australian and New Zealand Society of Neprhology Annual Scientific Meeting 2020; Tasmania Australia (Hobart: Virtual due to COVID-19).

